# Supplementary material for: Functionalized Mesoporous Silica as Doxorubicin Carriers and Cytotoxicity Boosters
Source: Nanomaterials (Basel). 2022 May 26;12(11):1823. doi: 10.3390/nano12111823 (PMC9182127; doi:10.3390/nano12111823)
Supplement: Supplementary file 1 [file nanomaterials-12-01823-s001.zip › nanomaterials-1704874-supplementary.pdf]

nanomaterials
MDPI

Supplementary Materials

Functionalized Mesoporous Silica as Doxorubicin Carriers and Cytotoxicity Boosters

Carmen Racles [1], Mirela-Fernanda Zaltariov [1,*], Dragos Peptanariu [2], Tudor Vasiliu [2] and Maria Cazacu [1]

[1] Department of Inorganic Polymers, "Petru Poni" Institute of Macromolecular Chemistry, 41A Aleea Gr. GhicaVoda, 700487 Iasi, Romania; raclesc@icmpp.ro (C.R.); mcazacu@icmpp.ro (M.C.)
[2] Centre of Advanced Research in Bionanoconjugates and Biopolymers, "Petru Poni" Institute of Macromolecular Chemistry, 41A Aleea Gr. GhicaVoda, 700487 Iasi, Romania; peptanariu.dragos@icmpp.ro (D.P.); vasiliu.tudor@icmpp.ro (T.V.)
* Correspondence: zaltariov.mirela@icmpp.ro

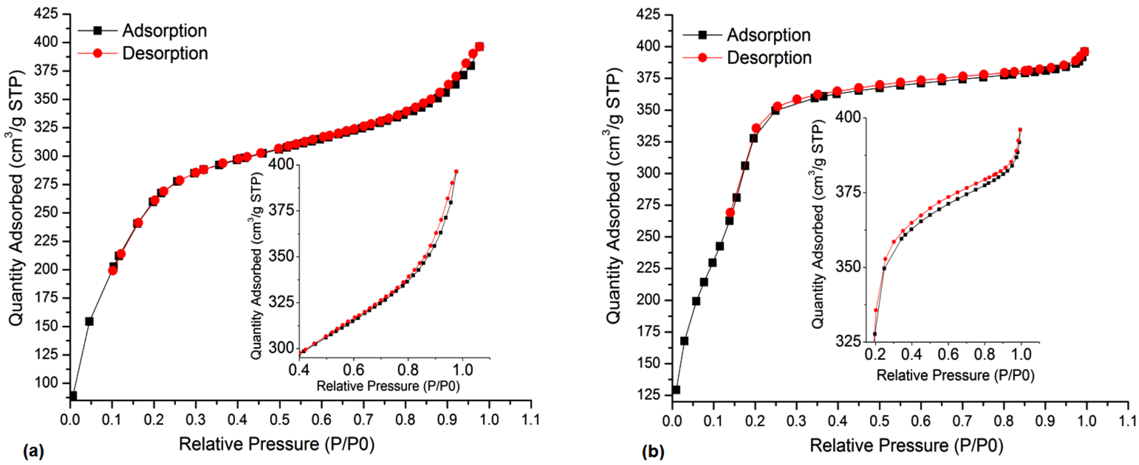

Figure S1. Nitrogen sorption-desorption isotherms of MSil1 (**a**) and MSil2 (**b**).

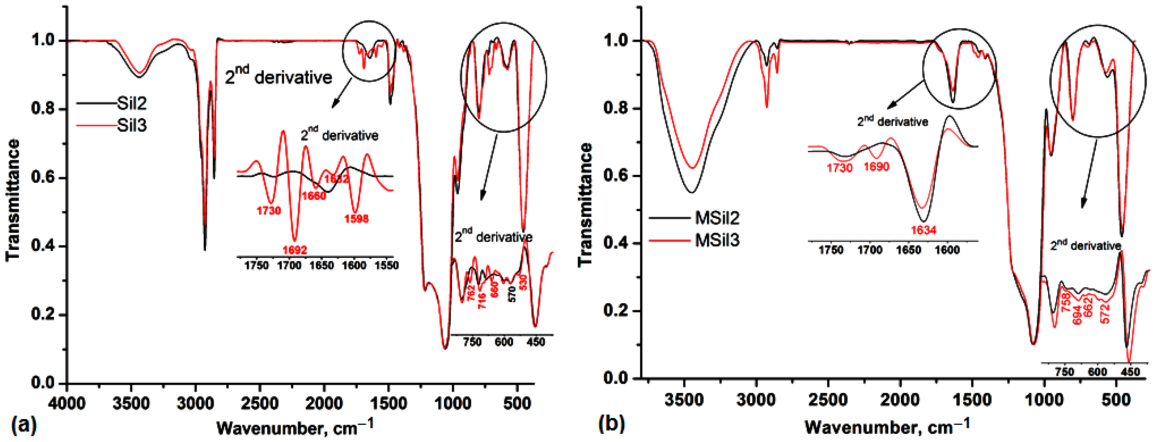

Figure S2. The FT-IR spectra of thiol functionalized silica: Sil2 (**a**) and MSil 2 (**b**)—black line and the glucose-modified silica: Sil3 (**a**) and MSil3 (**b**)—red line: the new bands due to glucose derivative and thioether are marked by 2nd derivative of the spectra in the 1750–1550 cm^{-1} and 800–400 cm^{-1}.

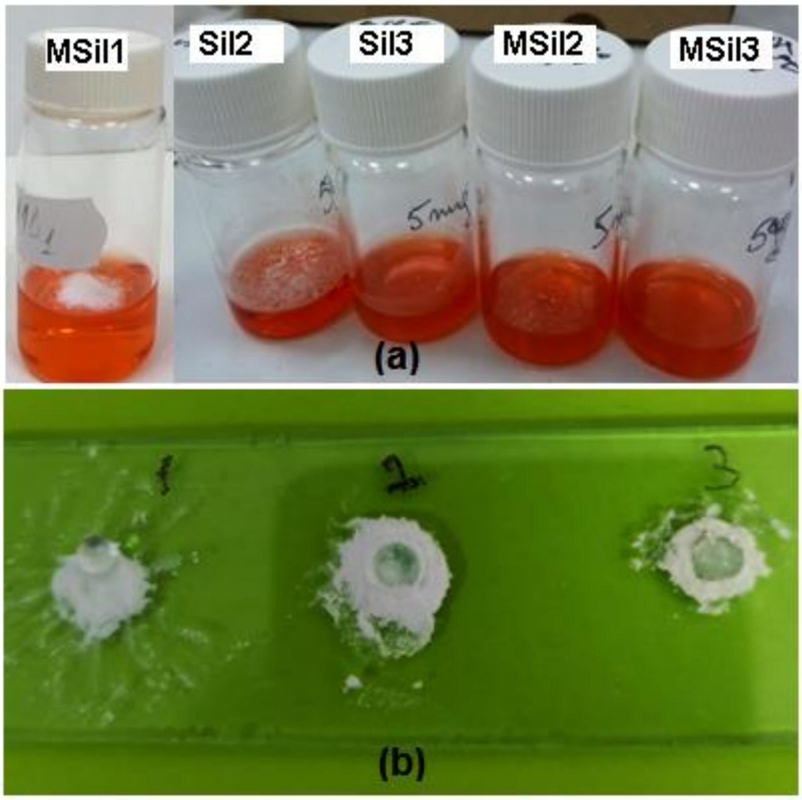

Code	Contact angle, °
MSil1	93.8 ± 2.3
MSil2	80 ± 1.8
MSil3	67 ± 1.2

(c)

Figure S3. Aspect of the silica powders at contact with an aqueous solution of doxorubicin hydrochloride (**a**), water drops placed on mesoporous silica MSil1-3 (**b**) and the water contact angles estimated from pictures of water droplets (**c**).

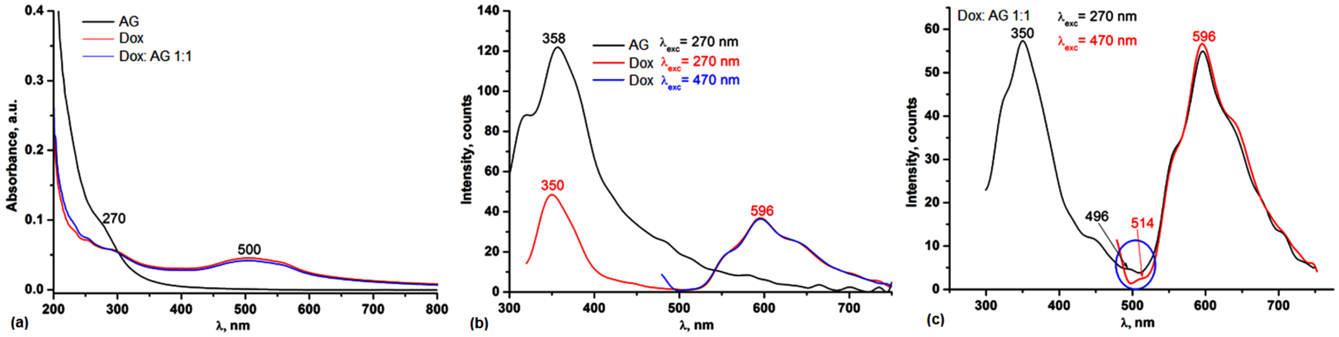

Figure S4. Absorption (**a**) and emission (**b**,**c**) spectra of AG, Dox and Dox:AG mixture 1:1.

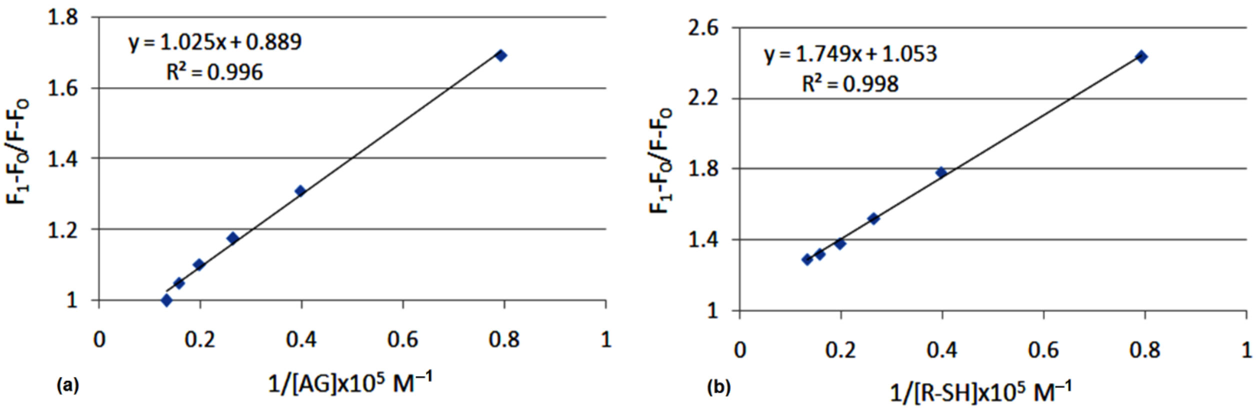

Figure S5. Benesi-Hildebrand plots for Dox: AG (**a**) and Dox: R-SH (**b**) complex formation, based on the fluorescence data (Figure 4).

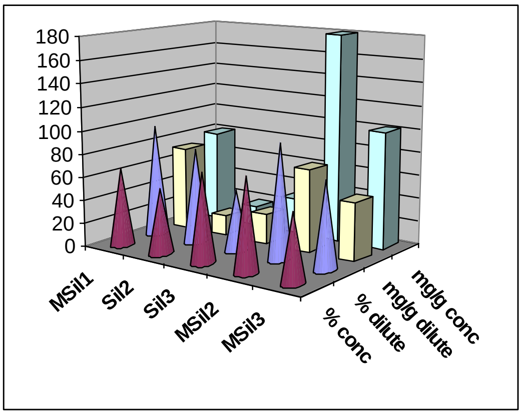

Figure S6. Maximum release at pH 5 from samples loaded in dilute and concentrated initial solution, expressed as % Dox released and amount of Dox released reported to silica.

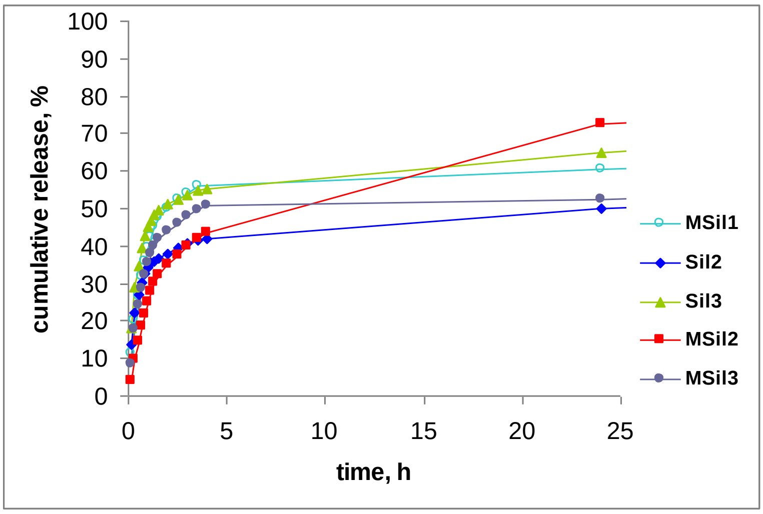

Figure S7. Dox release at pH = 5 (encapsulated at pH = 7.4 from 0.2% solution).

Table S1. Kinetics of Dox Release at pH= 5 from concentrated solution.

Sample	Higuchi		Korsmeyer–Peppas	
	K_H	R^2	n	R^2
MD1	29.71 (44.05; 13.48) [a]	0.9177 (0.9942; 0.9994)	0.503	0.9338
Sil2	14.92 (27.09; 7.27)	0.8209 (0.9265; 0.987)	0.309	0.8717
Sil84	20.02 (36.98; 7.67)	0.8212 (0.9541; 0.9868)	0.313	0.8815
MSil2	14.55 (35.07; 10.69) [b]	0.8781 (0.9995; 0.9942)	0.548	0.8681
MSil84	26.45 (40.67; 11.67)	0.8982 (0.9851; 0.9993)	0.537	0.899

[a] Two successive steps, before and after 90 min. [b] Kinetic data for 24h.

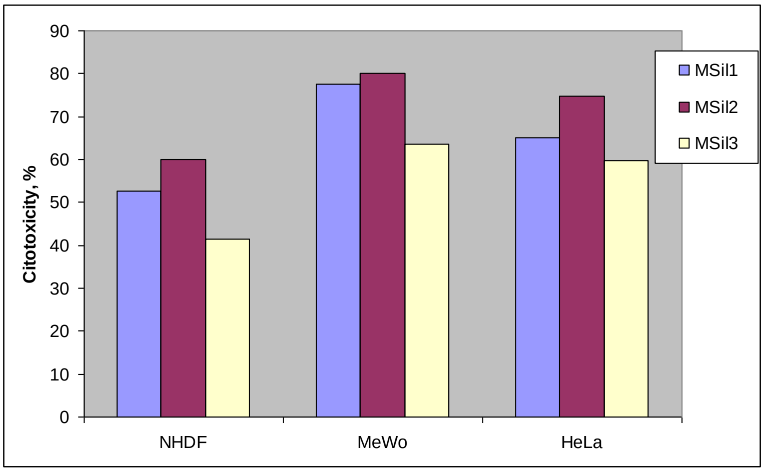

Figure S8. The net cytotoxic action of Dox, calculated as difference in cell viability in presence of Dox-free and Dox-loaded silica.
